# Evaluation of auto‐planning in IMRT and VMAT for head and neck cancer

**DOI:** 10.1002/acm2.12652

**Published:** 2019-07-04

**Authors:** Zi Ouyang, Zhilei Liu Shen, Eric Murray, Matt Kolar, Danielle LaHurd, Naichang Yu, Nikhil Joshi, Shlomo Koyfman, Karl Bzdusek, Ping Xia

**Affiliations:** ^1^ Department of Radiation Oncology Cleveland Clinic Cleveland OH USA; ^2^ Philips Healthcare Inc Fitchburg WI USA

**Keywords:** auto‐planning, head and neck, IMRT, step and shoot, VMAT

## Abstract

**Purpose:**

The purposes of this work are to (a) investigate whether the use of auto‐planning and multiple iterations improves quality of head and neck (HN) radiotherapy plans; (b) determine whether delivery methods such as step‐and‐shoot (SS) and volumetric modulated arc therapy (VMAT) impact plan quality; (c) report on the observations of plan quality predictions of a commercial feasibility tool.

**Materials and methods:**

Twenty HN cases were retrospectively selected from our clinical database for this study. The first ten plans were used to test setting up planning goals and other optimization parameters in the auto‐planning module. Subsequently, the other ten plans were replanned with auto‐planning using step‐and‐shoot (AP‐SS) and VMAT (AP‐VMAT) delivery methods. Dosimetric endpoints were compared between the clinical plans and the corresponding AP‐SS and AP‐VMAT plans. Finally, predicted dosimetric endpoints from a commercial program were assessed.

**Results:**

All AP‐SS and AP‐VMAT plans met the clinical dose constraints. With auto‐planning, the dose coverage of the low dose planning target volume (PTV) was improved while the dose coverage of the high dose PTV was maintained. Compared to the clinical plans, the doses to critical organs, such as the brainstem, parotid, larynx, esophagus, and oral cavity were significantly reduced in the AP‐VMAT (*P* < 0.05); the AP‐SS plans had similar homogeneity indices (HI) and conformality indices (CI) and the AP‐VMAT plans had comparable HI and improved CI. Good agreement in dosimetric endpoints between predictions and AP‐VMAT plans were observed in five of seven critical organs.

**Conclusion:**

With improved planning quality and efficiency, auto‐planning module is an effective tool to enable planners to generate HN IMRT plans that are meeting institution specific planning protocols. DVH prediction is feasible in improving workflow and plan quality.

## 1 INTRODUCTION

Head and neck (HN) cancer is a technically challenging treatment site in radiation oncology due to the complex anatomy and numerous organs at risk (OARs) in close proximity to targets. Treatment planning techniques for HN cancer have advanced from the conventional three‐field technique to intensity modulated radiation therapy (IMRT) over two decades.^1^ To achieve adequate target coverage while protecting numerous OARs, IMRT plans for HN cancer require highly conformal dose distributions and a steep dose fall‐off between the boundary of tumor volumes and sensitive structures. With limited clinical resources (time and manpower), a major challenge in HN IMRT planning is large variations in plan quality among treatment planners in part due to varied planning skills and limited planning time.^2^, ^3^, ^4^

Many publications have identified variations in IMRT plan quality. Hunt et al.^5^ quantified geometric factors that influenced dosimetric sparing of the parotid in IMRT plan for HN cancer in 2006. Moore et al.^3^ developed a model to predict the mean dose of an organ that overlaps with the planning target volume (PTV) and found that clinical implementation of this predictive model successfully reduced the plan variations. Wu. et al.^6^ and Yuan et al.^7^ built a knowledge‐based model to predict best achievable plan quality thus reducing plan quality variations. Knowledge‐based treatment planning, however, depends on the plan qualities that are used for model building and the specific clinical practice of how planning target volumes and prescription doses are defined and prescribed. Allowing flexibility and patient‐specific organ sparing prediction, a commercial product, PlanIQ Feasibility (Sun Nuclear Corp., Melbourne, FL), has been developed. The predicted dose volume histograms (DVHs) are based on energy‐specific dose spread calculation, reflecting the characteristics of photon dose distribution in media.^8^ Another approach to robust planning is to create many planning solutions (multicriteria optimization) for a single clinical case so that clinicians can make a decision based on the trade‐off among the dose coverage of the tumor volume and protections of sensitive structures.^9^ The automatic planning tool developed by the Pinnacle (Philips Radiation Oncology Systems, Fitchburg, WI) commercial treatment planning system is to mimic the manual processes of skilled planners by progressively and iteratively adjusting and adding planning objectives, which may mitigate the shortcoming of the gradient‐based optimization.^10^ In an ideal world, a planner would be equipped with all of these tools: a tool that can reliably predict achievable DVHs as initial inputs of the planning objectives, a tool that can automatically and progressively adjust planning objectives, and a tool that can offer multiple solutions based on different trade‐offs.

The purposes of this study are to (a) investigate whether the use of automation and multiple iterations can improve quality of HN plans; (b) determine whether delivery methods such as step‐and‐shoot (SS) and volumetric modulated arc therapy (VMAT) impact plan quality; (c) report on the observations of auto‐plan qualities with respect to the prediction of the feasibility tool.

## 2 MATERIALS AND METHODS

### 2.A Patient selection

Twenty HN patients with various tumor sites and stages were retrospectively selected from an institutional review board approved registry. We purposely chose these patients to reflect various clinical scenarios. The first ten of the twenty patient plans were used for initial testing of the auto‐planning model in Pinnacle. These patients were treated with nine‐beam step‐and‐shoot IMRT plans for either definitive or postoperative intent. For definitive cases, the primary targets were prescribed to a dose of 70–72 Gy while the regional lymph nodes were prescribed to a dose of 54–58 Gy. For post‐operative cases, the prescription doses to the primary tumor beds were 60–66 Gy. The details of the tumor locations, stages, and prescription doses for the second set of ten HN patients are listed in Table 1.

**Table 1:** Patient demographics.

Patient #	Tumor site	Stage	Prescription (Gy)
1	R BOT	T2N2cM0	72/58
2	Larynx & bilat LN	T3N2cM0	70/56
3	L BOT & bilat LN	T4aN2cM0	70/56
4	L tongue & L neck	T2N0M0	60/56
5	L tonsil & neck LN	T1N2bM0	66/56
6	R BOT & bilat LN	T3N2cM0	72/58
7	Oral cavity & bilat LN	T4aN2bM0	64/54
8	BOT bilat LN	T2N2bM0	70/56
9	Palate & bilat LN	T4aN0M0	70/56
10	BOT & bilat neck	T3N2cM0	70/56

R = right; L = left; BOT = base of tongue; bilat = bilateral; LN = lymph nodes.

### 2.B HN planning goals

The general HN planning goals and plan acceptance criteria have been established in our department. The treatment goals were to deliver prescription doses to ≥95% of the high dose planning target volumes (HD_PTV) and ≥95% of the low dose planning target volumes (LD_PTV). The planning acceptance criteria for OARs are listed in Table 2. Planners adjusted the planning goals for individual cases in consultation with the attending physicians due to variability in the anatomic relationship between PTVs and OARs across each case.

**Table 2:** Head and neck planning acceptance criteria.

Organs	Criteria
HD_PTV	V_Rx_ ≥ 95%
LD_PTV	V_Rx_ ≥ 95%
Spinal cord	D_max_ < 45 Gy
Brainstem	D_max_ < 54 Gy
Brainstem	V_30Gy_ < 50%
Contralateral parotid	D_mean_ < 26 Gy
Larynx	D_mean_ < 35 Gy
Mandible	D_max_ < 75 Gy
Trachea	D_mean_ < 45 Gy
Esophagus	D_mean_ < 50 Gy
Lips	D_mean_ < 20 Gy
Oral cavity	D_mean_ < 35 Gy
Submandibular glands	D_mean_ < 39 Gy

HD_PTV, high dose planning target volumes; LD_PTV, low dose planning target volumes.

### 2.C Auto‐planning module

A commercial auto‐planning module from the Pinnacle^3^ treatment planning system (Pinnacle^3^ 9.10, Philips Healthcare Inc., Fitchburg, WI) was clinically implemented in our institution in January 2015. Prior to clinical implementation, we validated this tool by comparing the second set of ten clinical HN plans to step‐and‐shoot IMRT plans and two‐arc volumetric modulated arc therapy (VMAT) plans generated from the auto‐planning module on the same patient data sets. All clinical plans used nine equally spaced beams, 6 MV photon energy, and step‐and‐shoot delivery method. The step‐and‐shoot IMRT auto‐plans (AP‐SS) used the same beam angles as the clinical plans. Since our practice has transitioned to VMAT delivery for most HN patients, we also compared two‐arc VMAT auto‐plans (AP‐VMAT) of these patients with their clinical plans.

In manual planning for HN cancer, typically more than 40 planning objectives and their associated numerical weights are entered by the planner. With the auto‐planning module, simplified planning goals as shown in Table 3 are entered by the planner. Based on the user input in the planning goals, the auto‐planning module then creates detailed planning objectives. The auto‐planning module uses an iterative process to mimic the manual planning process by separating overlapped contours, creating tuning structures, adjusting hot and cold spots, and optimizing conformality and homogeneity. After auto‐planning is completed, planners can further manually adjust the planning objectives and continue the “warm start” optimization as they often do during manual optimization. Or the planners can reset all beams, adjust planning goals, and start the auto‐planning process from the beginning. To further automate the treatment planning, users may create a site‐specific planning technique or a class solution, saved as a technique into the institution's library, which defines common planning parameters such as prescriptions, beam angles, beam energy, and treatment machine.

**Table 3:** Target and organs at risk optimization goals used in the auto‐planning technique for head and neck intensity modulated radiation therapy and volumetric modulated arc therapy.

ROI	Type	Dose (cGy)	Volume	Priority	Compromise
Brainstem	Max DVH	2000	1%	High	Checked
Cochlea_R	Max dose	400		Medium	Checked
Cochlea_L	Max dose	400		Medium	Checked
Pharynx	Max DVH	5600	50%	High	Checked
Pharynx	Mean dose	4000		High	Checked
Esophagus	Mean dose	2000		Medium	Checked
Larynx	Max DVH	5600	5%	Medium	Checked
Larynx	Mean dose	3000		High	Checked
Mandible	Max DVH	7000	1%	High	Checked
Oral cavity	Mean dose	3000		High	Checked
Parotid_R	Mean dose	2500		High	Checked
Parotid_L	Mean dose	2500		High	Checked
Spinal cord	Max dose	4200		High	Unchecked
Submandibular_R	Mean dose	3900		High	Checked
Submandibular_L	Mean dose	3900		High	Checked
Supraglottis	Mean dose	3000		High	Checked
Trachea	Mean dose	2500		Medium	Checked
Lips	Mean dose	2000		Medium	Checked
Lips	Max dose	3500		High	Checked

DVH, dose volume histograms; L, left; R, right.

For the purpose of this study, we created two HN specific techniques: one used the nine beam step‐and‐shoot delivery, and the other used two VMAT arc delivery. Both techniques used the same planning goals for normal structures. Since our institution uses multiple machines to treat HN patients and some treatment machines do not have VMAT delivery, planners still must choose a specific treatment machine after loading the HN specific planning technique.

For the nine‐beam AP‐SS, the direct machine parameter optimization (DMPO) was chosen and the two‐arc AP‐VMAT, the optimization type chosen was the “SmartArc” from the Pinnacle system with the dose calculation at every 4˚ with a convolution and superposition algorithm. For each HN case selected for this study, three plans were created: one clinical plan, one AP‐SS, and one AP‐VMAT.

### 2.D Plan evaluation

Plan quality was evaluated based on several dosimetric endpoints for PTVs and critical structures — including dose volume coverage, maximum dose to 0.03 cc (D_0.03cc_), and mean dose (D_mean_) — as well as the conformality index (CI), the homogeneity index (HI), and the total monitor units (MUs) per fraction. The CI^11^ was defined as

(1) $$CI=\frac{V_{\mathrm{Rx}}}{V_{\mathrm{PTV}}},$$

where V_Rx_ is the tissue volume covered by the prescription dose for the HD_PTV and V_PTV_ is the volume of the HD_PTV. For the ideal case, CI = 1. The HI was defined as

(2) $$HI=\frac{D_{\max}}{D_{\mathrm{Rx}}},$$

where D_max_ is the maximum dose of the plan and D_Rx_ is the prescription dose for the HD_PTV. The total MUs per fraction were also used to assess the plan delivery efficiency.

### 2.E Feasibility prediction

A commercial product, PlanIQ Feasibility (Sun Nuclear Corp. Melbourne, FL), output was compared with AP‐VMAT plan dosimetric endpoints. The PlanIQ predicts the best possible DVHs for each OAR, assuming PTVs are 100% covered by prescription dose. The *f* factor is defined as the feasibility factor, with higher feasibility associated with higher *f*. The estimation is based on a series of energy‐specific dose spread calculations, independent of any particular beam arrangement.^8^ For a specific patient, this estimated calculation is based on the heterogeneous dataset along with the geometric relationship between the targets and OARs while taking into account the high‐(penumbra driven) and low (PDD and scatter‐driven) gradient dose spreading. The predicted DVHs from PlanIQ can be used as the input of IMRT planning objectives or as a tool for quality assurance. In this paper, we use the predicted DVHs for the latter.

### 2.F Statistical analysis

One sided paired sign test was used to test the difference in medians of the dosimetric endpoints between the clinical plans and the corresponding AP‐SS and AP‐VMAT plans.^12^ The test is conducted by subtracting the paired values from two groups and counting the positive (*c+*) or negative (*c*−) signs. Let *c* equal the smaller one of *c+ *and *c*−, and let *N* be the total number of unequal pairs. The P‐value is given by the cumulative binomial distribution,

(3) $$P=\sum_{i=0}^{c}\left(\begin{array}{c} N\\ i \end{array}\right){\left(\frac{1}{2}\right)}^{N}.$$

The one sided test was used under the null hypothesis — the AP‐SS/AP‐VMAT plans are not better than the clinical plans in compared items. Statistical significance is achieved when *P* < 0.05 to conclude that the AP‐SS/AP‐VMAT plans are better than the clinical plans.

The Spearman rank correlation coefficient is used to describe the monotonic association between the PlanIQ feasibility and AP‐VMAT endpoints.^13^ The correlation coefficient is given by the following equation,

(4) $$r_{S}=1-\frac{6\sum_{i=1}^{N}\left(\Delta d_{i}^{2}\right)}{N\left(N^{2}-1\right)},$$

where Δ*d_i_* is the difference between the ranks for each pair, and *N* is the total number of pairs.

The correlation coefficients were interpreted as: very high correlation if *r_s_* > 0.9; high correlation if 0.7 < *r_s_* ≤ 0.9; moderate correlation if 0.5 < *r_s_* ≤ 0.7; low correlation if 0.3 < *r_s_* ≤ 0.5; negligible correlation if *r_s_* ≤ 0.3.

## 3 RESULTS

**Figure 1:**
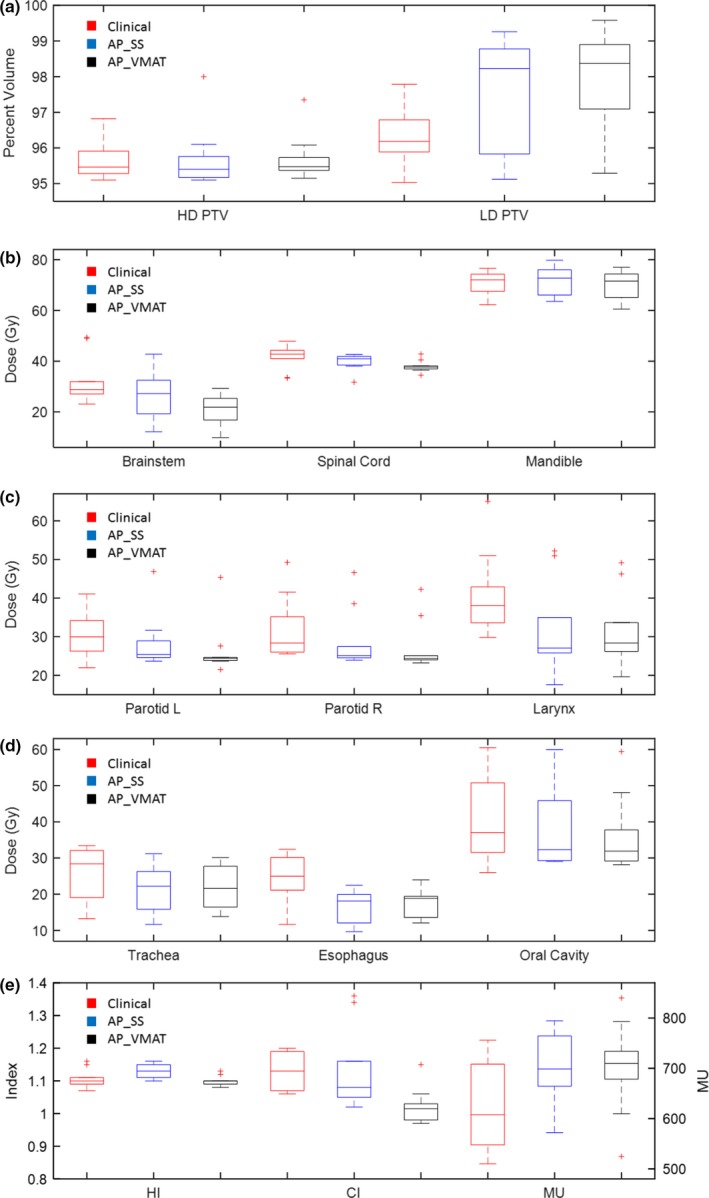
Comparison of dosimetric endpoints, HI, CI, and MU, of the clinical, AP_SS, and AP_VMAT plans for the ten HN patients. Results are depicted with box plots with median, interquartile range (IQR), minimum, and maximum values. Outliers are marked in red crosses (“+”). (a) Percent volume of high dose and low dose PTV covered by the prescription doses; (b) D_0.03cc_ of brainstem, spinal cord, and mandible; (c) Mean dose of parotids and larynx; (d) Mean dose of trachea, esophagus, and oral cavity; (e) Homogeneous index, conformity index, and monitoring units.

Figure 1 shows the median, interquartile range (IQR), minimum, and maximum values of the selected dosimetric endpoints, HI, CI, and MU for the second set of ten HN patients. All AP‐SS plans and AP‐VMAT plans met the clinical dose limit requirements. As shown in Table 4, with the use of the AP‐VMAT, the coverage of LD_PTV significantly improved from that of the clinical plans (98.37% vs 96.18%; *P* = 0.001) while the same dose coverage of the HD_PTV was maintained. The doses to critical structures, such as the brainstem, parotids, larynx, esophagus, and oral cavity, were significantly reduced in the AP‐VMAT plans (*P* < 0.05). Compared with the clinical plans, the AP‐SS plans had significantly lower doses to the spinal cord, larynx, and esophagus (*P* < 0.05). Both AP‐SS and AP‐VMAT plans had better or similar homogeneity and conformality, and higher MU than that of clinical plans.

**Table 4:** Plan quality endpoints of the Clinical, AP_SS and AP_VMAT plans. One sided paired sign tests were performed between the Clinical and AP_SS, and between the Clinical and AP_VMAT. Results with *P* < 0.05 indicated statistical significance and were labeled with “*”.

	Clinical		AP_SS			AP_VMAT		
	median	IQR	median	IQR	p‐value	median	IQR	p‐value
HD PTV coverage	95.47%	0.55%	95.40%	0.54%	0.377	95.47%	0.30%	0.623
LD PTV coverage	96.18%	0.79%	98.23%	2.46%	0.055	98.37%	1.59%	0.001*
Brainstem D_0.03cc_	28.77	4.72	27.26	10.88	0.055	21.85	8.37	0.011*
Spinal Cord D_0.03cc_	42.76	2.97	40.93	3.07	0.011*	37.66	0.96	0.055
Mandible D_0.03cc_	72.06	6.61	72.74	9.12	0.623	71.56	8.31	0.377
Parotid left mean	29.95	7.14	25.35	3.41	0.172	24.37	0.74	0.011*
Parotid right mean	28.37	7.39	25.12	2.82	0.055	24.35	0.98	0.011*
Larynx mean	38.06	5.42	27.04	3.63	0.020*	28.36	2.85	0.002*
Trachea mean	28.40	11.62	22.21	9.04	0.055	21.63	9.96	0.055
Esophagus mean	24.97	7.75	18.14	5.99	0.011*	18.86	5.77	0.011*
Oral cavity mean	37.03	16.66	32.27	14.06	0.172	31.91	8.29	0.011*
HI	1.10	0.03	1.13	0.03	0.055	1.10	0.01	0.623
CI	1.13	0.10	1.08	0.09	0.172	1.02	0.04	0.011*
MU	607.75	144.22	698.81	95.28	0.055	709.60	51.06	0.623

AP_SS, step‐and‐shoot auto‐plan; AP_VMAT, volumetric modulated arc therapy auto‐plan; CI, conformality indices; HD, high dose; HI, homogeneity indices; IQR, interquartile range; LD, low dose; MU, monitor units; PTV, planning target volume.

**Figure 2:**
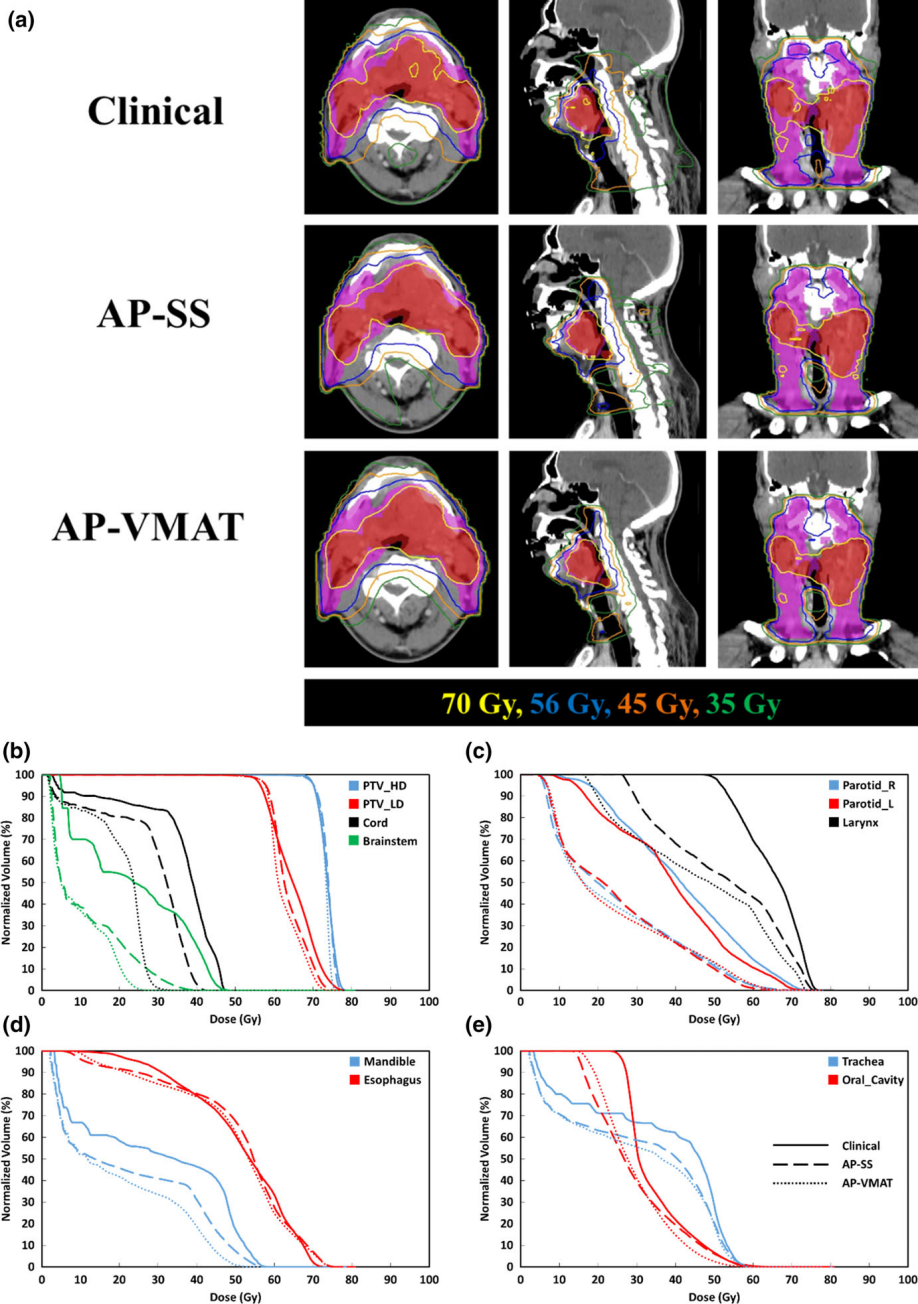
(a) Dose distributions represented by isodose lines from the nine‐beam step‐and‐shoot clinical plan, the nine‐beam step‐and‐shoot auto‐plan (AP_SS), and the two‐arc volumetric modulated arc therapy auto‐plan (AP_VMAT). The red colorwash is the planning target volume (PTV_70) and the magenta colorwash is the PTV_56. (b–e) Dose volume histograms of the clinical (solid), AP‐SS (dash), and AP‐VMAT (dot) plans for the PTVs and organs at risks (OARs).

For a selected patient, Fig. 2(a) shows the dose distributions of the clinical, AP‐SS, and AP‐VMAT plans. This patient received 70 Gy to the primary tumor and 56 Gy to the regional lymph nodes. The isodose lines are more conformal to the tumor volumes in the AP‐VMAT plan compared to the clinical step‐and‐shoot and the AP‐SS plans, especially in the sagittal views. Figures 2(b)‐2(e) show the DVHs of the three plans for this patient. All three plans have similar coverage of the PTV_70, while both AP‐SS and AP‐VMAT have slightly better coverage of PTV_56 compared to the clinical plan [Fig. 2(b)]. For critical structures such as the brainstem, spinal cord, larynx, right parotid, esophagus, and trachea, the doses in both AP‐SS and AP‐VMAT plans are lower than the clinical plan [Figs. 2(c)2(e)]. For several structures such as the spinal cord, larynx, and esophagus, the AP‐VMAT plan achieved even lower doses than the AP‐SS plans. This indicates Pinnacle^3^'s auto‐planning module is able to lower doses to critical structures while maintaining PTV coverage.

**Figure 3:**
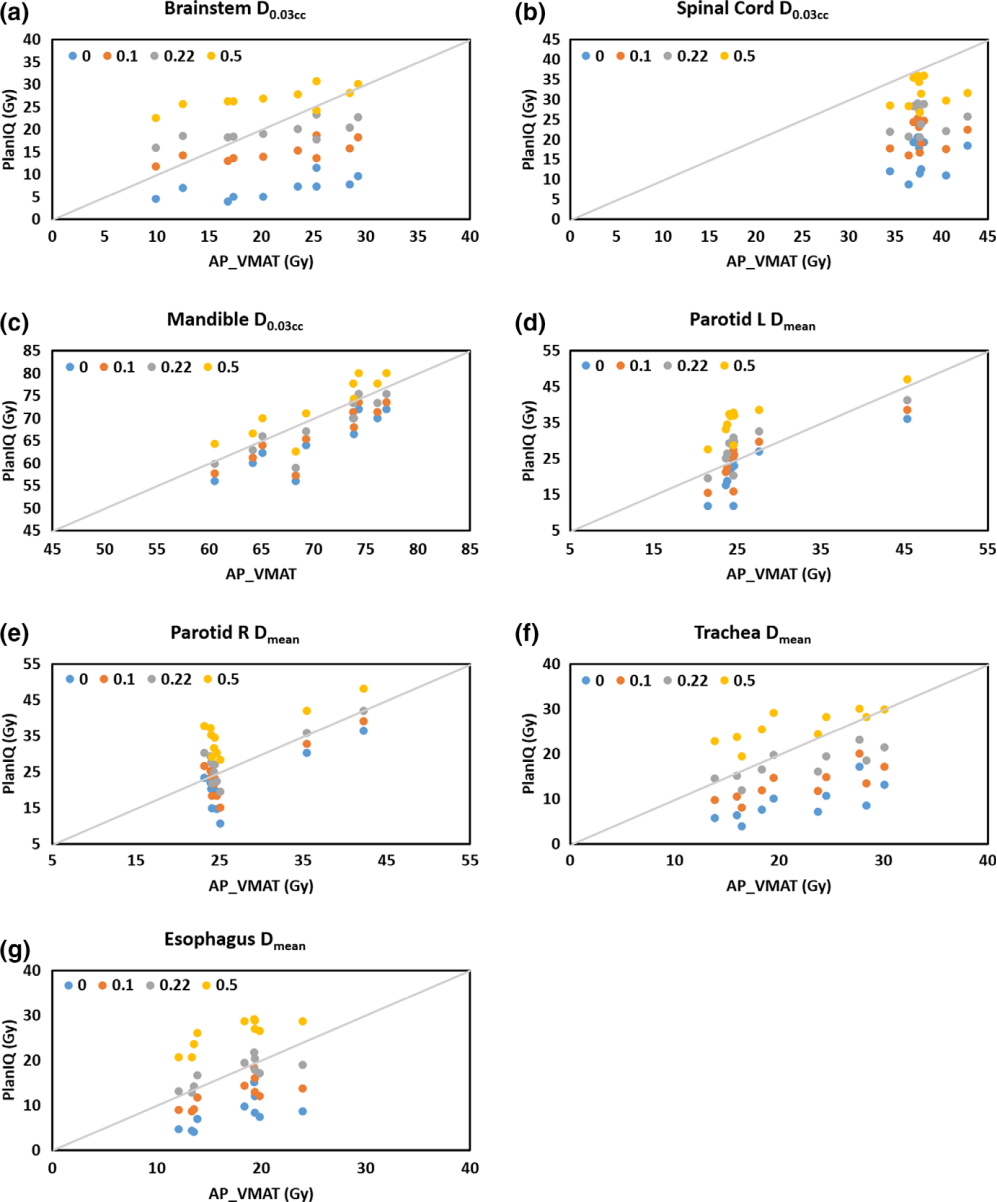
Scatter plots of PlanIQ predictions with *f* = 0, 0.1, 0.22, and 0.5 against volumetric modulated arc therapy auto‐plan (AP_VMAT) endpoints for (a) brainstem D_0.03cc_, (b) spinal cord D_0.03cc_, (c) mandible D_0.03cc_, (d) left parotid D_mean_, (e) right parotid D_mean_, (f) trachea D_mean_, and (g) esophagus D_mean_. A *y = x* reference line is displayed in each figure representing a perfect prediction.

With four different *f* factors (*f* = 0, 0.1, 0.22, and 0.5), the PlanIQ predicted OAR dosimetric endpoints were compared to that of the AP‐VMAT plans. Table 5 lists the correlation coefficients between the predicted and achieved plans. High correlations are observed for D_0.03cc_ to brainstem and mandible, and D_mean_ to left parotid, and trachea. Negligible correlations are observed for D_0.03cc_ to spinal cord and D_mean_ to right parotid. The results are also displayed in Fig. 3, where the PlanIQ prediction with each *f* factor was plotted against the AP‐VMAT endpoint. Perfect prediction is reflected as a theoretical *y = x* line, which is displayed in each figure. High correlation between predictions and planned results is shown as a monotonic trend as well as seen in the proximity to the *y = x* line.

**Table 5:** Correlation coefficients between PlanIQ feasibility and volumetric modulated arc therapy auto‐plan endpoints with *f* = 0, 0.1, 0.22, and 0.5.

Endpoint	*f* = 0	*f* = 0.1	*f* = 0.22	*f* = 0.5
Brainstem D_0.03_	0.817	0.721	0.649	0.681
Cord D_0.03_	0.097	0.103	0.152	0.176
Mandible D_0.03_	0.899	0.878	0.878	0.878
Parotid left D_mean_	0.760	0.790	0.782	0.733
Parotid right D_mean_	0.030	0.067	0.067	0.067
Trachea D_mean_	0.818	0.818	0.794	0.794
Esophagus D_mean_	0.620	0.657	0.657	0.697

## 4 DISCUSSION

This study shows the feasibility of generating clinically acceptable plans using the Pinnacle auto‐planning module. PTV dose coverage was similar or improved while the doses to critical structures were decreased beyond the desired dose limits. A challenge of HN IMRT planning is the use of mathematical objectives to describe a spatial dose distribution. To mitigate the lack of spatial information in planning objectives, the number of contours and associated dose constraints are increased. With more than 40 sensitive structures defined, it is challenging for planners to manually adjust the numerical weighting factor for each structure, resulting in large variations of plan quality. In initial clinical plans (manual plans), the dose limits to the critical structures such as the spinal cord, brainstem, and parotid glands are met but the dose limits to other noncritical structures such as oral cavities, larynx varied greatly.

Another challenge for HN planning is the use of gradient search optimization and the presence of the nonconvex problem in inverse planning for radiotherapy. Therefore, previous planning experience is difficult to generalize for complex plans of head and neck cancer. For example, during IMRT planning, planners do not start to include all planning objectives at once but add planning objectives in a piecemeal fashion. Frequently, planners include artificial tuning structures such as ring structures or structures of cold or hot spots based on optimized results. Because of this progressive process, final planning objectives for the same patient cannot be reused to reproduce the same plan quality. The auto‐planning module automates this progressive planning process. During manual planning for a typical HN case, there are often two to three PTVs with different prescription doses and 20 to 40 sensitive structures with different dose constraints and importance weights. With manual planning, setting optimal dose constraints of these numerous sensitive structures is time consuming. A planner may stop the iterative optimization before achieving an optimal plan provided that the major dosimetric constraints for the critical structures are met. Auto‐planning is able to systematically add many more planning objectives and artificial contours than those of a typical planner could, resulting in the improvement of plan quality.

The purposes of using the automatic planning tool are not only to improve plan quality but also to improve planning efficiency. With auto‐planning, a planner can create acceptable clinical quality plans within a restricted time. One launch of the auto‐planning process takes approximately 15 min for fixed gantry step‐and‐shoot delivery and approximately 1 h for VMAT delivery. These estimated times may vary depending on the available computational power, dose grid volume and resolution, and number of beams. The auto‐planning module optimizes the plan without the need for the planner to consistently monitor optimization progress and manually modify optimization objectives. It is difficult to directly compare the computation time between auto and manual planning since human time and machine time weigh differently. Overall, auto‐planning requires longer computation time but saves human time significantly. While different optimization engines are used, Vanderstraeten et al. show that for lung stereotactic body radiotherapy auto‐planning reduces optimization time by 77.3% and total monetary cost by 3.6%.^14^ As reported by Creemers et al., auto‐planning requires roughly the same total planning time compared to manual planning, but it reduces the planners' “hands‐on‐time” by 75%.^15^

The auto‐planning module has some limitations. The beam arrangement must be initially set and cannot be changed during auto‐planning. Auto‐planning runs six optimization iterations, which may be excessive for simple cases. Though auto‐planning techniques used in this study generated clinically acceptable plans for all ten HN patients without further modification, other patient cases may still require manual adjustments to achieve optimal results.

In this work, nonparametric statistical tests such as sign test and Spearman rank correlation are used due to the small sample size. One must cautiously interpret the results as they are not as powerful as parametric tests such as *t*‐test and Pearson correlation. We study plans with two prescription dose levels to maintain the data homogeneity. However, three dose level HN plans are also common at other institutions while our institution has adopted to two dose levels for most patients with HN cancer. With more prescription levels, the geometric and dosimetric relationships between targets and OARs will change, which may affect the auto‐planning. Adjustment in the auto‐planning technique is needed to accommodate such prescription changes even for the same disease site.

Auto‐planning, among other methods such as knowledge‐based planning and multicriteria optimization, is one of the advanced planning techniques to improve planning consistency and efficiency. HN cancer is one of the most challenging sites for treatment planning, and Pinnacle auto‐planning is confirmed as a viable solution in an early study.^16^ Other sites, such as prostate,^17^ esophagus,^18^ lung,^14^, ^15^ and brain,^19^ are also investigated by different groups. All studies have confirmed that auto‐planning generates clinically acceptable plans. While auto‐planning is a potential solution to achieve good plan quality and standardization, an independent plan quality check tool is necessary. Combining knowledge‐based plan quality check and auto‐planning is one solution. Using a model trained with prostate plans, Janssen et al.^20^ demonstrate that knowledge‐based prediction detects 25% of the examined auto‐plans as suboptimal. Another solution, as discussed in this study, is to use a patient‐specific and anatomy‐driven DVH prediction tool. The latter has the advantage of not being dependent on past planning experience.

The PlanIQ predictions are performed independently to help evaluate plan quality in this study. PlanIQ may also be used before treatment planning to help guide the planning process. Fried et al. show that with the knowledge of PlanIQ predictions before HN planning, significant reduction in doses to contralateral parotid and larynx is achieved.^21^

Moderate or high correlations are observed in all ten AP‐VMAT plans and the corresponding PlanIQ predictions for five of seven OARs. To produce the exact predictions achievable by auto planning, the *f* factor must be adjusted. Further investigation with larger data sets is needed to determine the preferable *f* factors per treatment site based on institution‐specific planning requirements. PlanIQ predictions and AP‐VMAT show high correlation in the maximum dose to the brainstem but negligible correlation in the maximum dose to the spinal cord. They also show high correlation in the mean dose to the left parotid but negligible correlation in the mean dose to the right parotid. The authors speculate that the reliability of a certain *f* factor predicting DVH for each OAR depends on the location of the tumor, for example, location in the superior‐inferior direction, lymph node level, left or right, unilateral or bilateral.

As shown in Figs. 3(d) and 3(e), while the predicted mean dose to the parotids has a range, the AP‐VMAT plans mostly cluster around 25 Gy, which is reflective to the clinical requirements (Table 2) and auto‐planning technique (Table 3). Although the AP‐VMAT plans do not meet the predicted spinal cord dose [Figure 3(b)], they all meet the clinical requirements, and they are compromised so that other OARs like the parotids also meet the requirements. The feasibility prediction assumes isotropic dose fall off rate surrounding the target, while in reality dose is designed to fall off differentially based on the importance and difficulty of the OAR constraints in each direction.

## 5 CONCLUSIONS

Nine‐beam step‐and‐shoot and two‐arc VMAT treatment planning techniques are developed using Pinnacle auto‐planning for HN conventional radiotherapy. This auto‐planning tool is promising in reducing clinical workload and improving plan quality. DVH predictions with PlanIQ feasibility show good agreement with AP‐VMAT plans in the initial testing. Further study is warranted in order to fully implement the prediction tool for clinical use.

## CONFLICT OF INTEREST

Karl Bzdusek is an employee of Philips Healthcare Inc. Ping Xia received a research grant from Philips Healthcare Inc. Others: None.
